# Application of block matching method-based Echocardiography combined with serum NT-PROBNP level detection in the early prediction of PDA in premature infants

**DOI:** 10.12669/pjms.37.6-WIT.4864

**Published:** 2021

**Authors:** Chunying Wang, Yunlong Shi, Jianwei Ji

**Affiliations:** 1 Chunying Wang, attending physician. Department of Neonatology, Yiwu Central Hospital, Yiwu, 322000, China; 2 Yunlong Shi, attending physician. Department of Neonatology, Yiwu Central Hospital, Yiwu, 322000, China; 3 Jianwei Ji, attending physician. Department of Neonatology, Yiwu Central Hospital, Yiwu, 322000, China

**Keywords:** Serum NT-PROBNP, patent ductus arteriosus, Premature Infants, Block Matching Method-Based Echocardiography

## Abstract

**Objectives:**

The paper uses block matching method combined with echocardiography to explore the value of N-terminal pro-brain natriuretic peptide (NT-proBNP) in predicting symptomatic patent ductus arteriosus (PDA) in preterm infants.

**Methods:**

We selected premature infants born between February 2019 and March 2020, gestational age ≤32 weeks, and echocardiography within 48 hours to determine the presence of arterial ducts as the research object, monitor their clinical manifestations, and detect serum at three and five days after birth The level of NT-proBNP was checked with echocardiography, and the children were divided into PDA group and asymptomatic patent ductus arteriosus (aPDA) group according to the clinical manifestations and echocardiographic.

**Results:**

The area under the ROC curve of PDA predicted by serum NT-proBNP level at 3 days after birth was 0.949, the cut-off value was 27035pg/mL, the sensitivity was 92.3%, and the specificity was 94.6%; serum NT-proBNP level at 5 days after birth predicted the ROC curve of PDA The lower area is 0.924, the critical value is 6411 pg/mL, the sensitivity is 92.3%, and the specificity is 92.9%.

**Conclusion:**

NT-proBNP may be a quantitative indicator of arterial duct shunt; the detection of serum NT-proBNP levels at 3 and 5 days after birth is helpful for early prediction of PDA.

## INTRODUCTION

Patent ductus arteriosus (PDA) is one of the common complications of preterm infants and it is particularly important to predict the emergence of PDA early [1]. Echocardiography is the gold standard for diagnosing PDA, but the equipment is expensive and cannot be widely used in primary hospitals [2]. This study prospectively analyzed the clinical course of premature infants with PDA, while studying the correlation between NT-proBNP and echocardiographic indicators, as well as the sensitivity and specificity of NTproBNP in predicting PDA, and exploring the clinical value of early NT-proBNP levels in predicting PDA.

## METHODS

Premature infants who were born from February 2019 to March 2020 and were admitted in our hospital were the research objects.

### Inclusion criteria

(1) Admission within 24 hours after birth; (2) Gestational age ≤ 32 weeks; (3) Within 48 hours after birth, the presence of an arterial duct was confirmed by echocardiography.

### Exclusion criteria

(1) congenital heart disease other than PDA and patent foramen ovale; (2) sepsis; (3) neonatal persistent pulmonary hypertension; (4) asphyxia; (5) renal failure; (6) and Death cases not related to PDA.

The children were divided into PDA group and asymptomatic PDA (aPDA). The diagnostic criteria for PDA are as follows:

### (1) Clinical and chest radiograph indicators

Worsening respiratory conditions (shortness of breath and increased inhaled oxygen concentration, or mechanical ventilation required); continuity or systolic murmur can be heard above the left edge of the sternum; water pulse or precordial pulsation is obvious ; Blood pressure is difficult to maintain a normal level; Chest radiograph imaging shows: lung congestion or heart enlargement caused by pulmonary blood increase (cardiothoracic ratio greater than 60%).

### Ultrasonic indicators

Echocardiography found that there is a left-to-right shunt in the diastolic arterial duct; the duct is larger than 1.5mm and the ratio of the inner diameter of the left atrium to the inner diameter of the aortic root (LA/AO)>1.3. Those who meet the clinical and chest radiograph indicators 2 or more, and meet the ultrasound indicators are diagnosed as PDA.

### Data collection

(1) General information: gestational age, birth weight, gender, date of birth, time of admission, birth method, Apgar score, etc.; observe whether the research subjects have neonatal respiratory distress syndrome and neonatal necrotic small intestine during hospitalization Colitis, intraventricular hemorrhage, pneumonia, record the amount of fluid before the diagnosis of PDA, and whether pulmonary surfactants have been used.

### (2) Clinical symptoms:

deterioration of respiratory conditions (shortness of breath and increased inhaled oxygen concentration, or the need for mechanical ventilation); heart murmur; water pulse or precordial pulsation; blood pressure. (3) Chest radiograph: Perfect chest imaging examination according to clinical needs, and record whether there are signs of pulmonary blood increase and the ratio of cardiothoracic.

### Echocardiography

(1) For premature infants who were admitted within 24 hours and had a gestational age of ≤32 weeks, check echocardiography within 48 hours after birth to determine whether there is an arterial duct; (2) Check echocardiography for the enrollees 3 and 5 days after birth to check the indicators Including: arterial catheter diameter, arterial catheter blood flow shunt direction, LA/AO ratio, left ventricular end diastolic anteroposterior diameter (LVEDD), left ventricular end systolic anteroposterior diameter (LVESD), left ventricular ejection fraction (LVEF); (3) When premature infants have two or more PDA clinical and chest radiograph indicators, check echocardiogram again, record the diameter of the arterial catheter and the LA/AO ratio to confirm the diagnosis of PDA.

### Detection of serum NT-proBNP content

Take 1 mL of venous blood while checking the echocardiogram on the three and five days after birth, and centrifuge to collect the serum in a desiccant tube. Use the minivoid’s analyzer (detection range of 15~25000pg/mL) to detect NT-proBNP. When NT-proBNP>25000pg/mL, use the R1 solution provided in the kit to dilute 4 times, and then retest.

Image restoration method based on sample block matching When calculating data items, the main strategy is to propose different structural representation methods as much as possible, in order to effectively represent the structural components of the area to be repaired, and guide the restoration process along the main structural area in the image.^3^ However, due to the existence of strong texture components in many images, the repair results are sometimes not ideal^4^ therefore, the structural components of the image can be extracted first, and the repair process can be guided based on the structural components.

***Step1***. Establish the Gaussian pyramid □Gl□ of the image I, which is used to determine the parameters of the Step2 nonlinear mapping function.

***Step2***. According to the coefficients of Gaussian pyramids of different scales, the input image is transformed by point-by-point nonlinear mapping to generate multiple images after mapping. The mapping function is


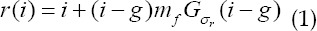


Among them, i is the pixel value of the original image; g is the Gaussian pyramid coefficient established at the corresponding position Step1; *σ_r_* is the contour discrimination threshold; 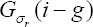 is the standard Gaussian function; *m_f_* is an amplitude factor of the mapping function. For g values of different scales, that is, different mapping images can be obtained.

***Step3.*** Calculate multiple processing results according to Step2, and calculate the coefficient of the output Laplacian pyramid {L_l_}.

***Step4***. Calculate the output result through the collapse of Laplacian Pyramid {L_l_}. The original image reconstructed by the collapse of Laplacian Pyramid is completed by recursively calling *G_l_=L_l_+u(G_l+1_)* (from the highest scale to the lowest scale, 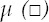 is the up sampling function, until *G_0_=I* is (Complete the original image reconstruction). The specific formula is


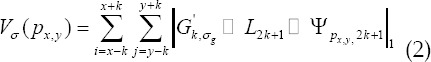


Among them, □ represents the matrix elements are multiplied point by point, 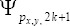 represents the image block with pixel point *p_x,y_* as the center and width and height 2*k*+1.


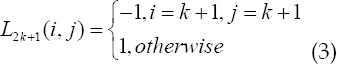


The extraction of image structure components can be achieved by smoothing the edges of the image. The adaptive selection of parameter *σ_r_* should be inversely proportional to the corresponding value in the local change *V_σ_* obtained by equation (3). Therefore, you can pass


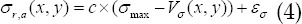



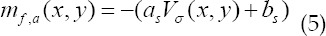


Obtain the adaptive threshold parameter matrix *σ_ra_* and the adaptive factor parameter matrix *m_f,a_*. They can be substituted into equation (4) to achieve the smoothing of the original image edge preservation details.

### Statistical Analysis

SPSS20.0 statistical software was used for analysis. The Wilcoxon rank sum test was used for comparison between groups; count data were described by the number of cases and percentage (%), and the comparison between groups was performed by chi-square test or Fisher’s exact probability method; the relationship between NT-proBNP levels and ultrasound indicators was analyzed by bivariate correlation analysis; ROC was used The curve evaluates the predictive value of NTproBNP on PDA and determines the best cut-off value; P<0.05 is considered statistically significant.

## RESULTS

### General Information

The 69 preterm children’s general information is given in Table-I.

**Table-I:** Comparison of general information, supportive treatment and complication rate between PDA group and aPDA group.

*Project*	*aPDA group*	*PDA group*	*t*	*p*
Gestational age	30.8±1.4	30.0±1.7	1.913	0.06
Birth weight	1454±265	1367±249	1.076	0.286
Boy/girl	32/24	9/4	(0.639)	0.424
Method of Birth (Cistern Delivery/Cesarean Section)	30/26	8/5	(0.271)	0.603
Necrotizing enterocolitis [n (%)]	3(5)	0(0)	-	1.000
Intraventricular hemorrhage [n (%)]	2(4)	2(15)	-	0.158
Respiratory distress syndrome [n (%)]	41(73)	9(69)	-	0.742
Pneumonia [n (%)]	24(43)	7(54)	(0.515)	0.473
Liquid intake (mL/kg per day)	98.7±1.8	95.7±2.4	0.775	0.441
Use of alveolar surfactant [n (%)]	33(59)	10(77)	-	0.343

### Analysis of clinical data in PDA group

In the PDA group, 13 premature infants showed early signs of PDA (precordial murmur, obvious precordial pulsation, worsening of breathing, rapid heart rate, etc.) 2-7 days after birth (average 3.7 days), 7-16 days after birth (Average 11d) was diagnosed as PDA. The clinical symptoms of 11 children were improved after interventional treatment with ibuprofen, of which 7 cases (64%) had closed arterial ducts, and the other 4 cases (36%) had reduced arterial duct diameters.

### Correlation analysis between NT-proBNP and ultrasound indicators

3d after birth serum NT-proBNP value and the arterial catheter diameter highly correlated (r = 0.856, P0.05). 5d after birth serum NT-proBNP value and the arterial catheter diameter, LA / AO ratio showed a significant positive correlation (respectively r = 0.528,0.721, both P0.05).

### Comparison of serum NT-proBNP value between PDA group and aPDA group

3d and 5dPDA after birth serum NT-proBNP was higher than aPDA group was statistically significant (P <0.001) differences in Table-II.

**Table-II:** Comparison of serum NT-proBNP levels between PDA group and aPDA group 3 and 5 days after birth.

*Group*	*Number of cases*	*3d after birth*	*5d after birth*
aPDA	56	10068±1361	3116±665
PDA	13	48539±8114	19713±5730
Z value	Number of cases	-5.018	-4.742
P value		<0.001	<0.001

### ROC curve analysis

**Fig.1:**
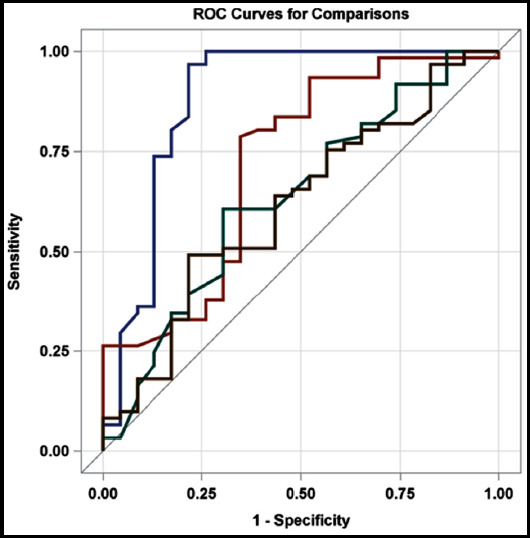
ROC curve of NT-proBNP to diagnose PDA

The area under the ROC curve of serum NT-proBNP 3d postnatal diagnosis PDA is 0.949 (95% CI: 0.892 ~ 1.000, P <0.001), the critical value 6411pg / mL, sensitivity of 92.3% and a specificity of 92.9% (Fig.1).

## DISCUSSION

Sixty-nine cases of premature infants were selected in this study. Echocardiography was performed after birth. Then, block matching method was used to process the ultrasonic images of the patients, and venous blood was collected to detect the level of NT-proBNP in the blood. The early ultrasonographic characteristics of PDA and the difference of serum NT-proBNP levels in premature infants were analyzed, and some research results were obtained. The results showed that among the 69 preterm infants in this study, 13 preterm infants developed early signs of PDA within 2-7 days after birth, such as precardiac murmur, increased breathing, and accelerated heart rate, and were all diagnosed with PDA within 7-16 days after birth. It suggested that there was a risk of delay in the clinical diagnosis of PDA, which in turn increased the probability of neonatal lung disease and chronic bronchopneumonia and prolonged the use of ventilator.^5^-^7^ Therefore, the diagnosis of PDA only based on neonatal ultrasound imaging findings may reduce the diagnostic sensitivity, which was similar to the findings of Lee.^8^ Clinical symptoms can’t reflect the changes of neonatal cardiac load in time, and it is difficult to provide clinical basis for the early treatment of PDA. After ibuprofen interventional treatment, ductus arteriosus closure was detected in 7 cases, ductus arteriosus diameter was reduced in 4 cases, and no significant improvement was found in the remaining 2 cases. Neonatal arterial diameter and LA/AO ratio are two important indicators for evaluating ductal shunt and hemodynamics of neonatal PDA. The results showed that the level of NT-proBNP in neonatal serum was positively correlated with the diameter of ductus arteriosus and the ratio of LA/AO, which was similar to the research results of Alenazi.^9^ The serum level of NT-proBNP was highly correlated with the diameter of ductus arteriosus at 3 days after birth. The serum level of NT-proBNP was positively correlated with the diameter of ductus arteriosus and the ratio of LA/AO at 5 days after birth. It may be caused by the left to right atrial shunt caused by PDA in neonatal patent foramen ovale at three days of birth, reducing the volume load of the left atrium.^10^ These results suggested that neonatal serum NT-proBNP level can better reflect the changes of cardiac volume load than clinical symptoms, and may be a quantitative indicator of arterial shunt, which can reflect the hemodynamic changes of neonates with PDA. The serum NT-proBNP values of the two groups of neonates were analyzed, and it was found that the serum NT-proBNP in the PDA group was significantly higher than that in the APDA group after 3d and 5d of birth, which was similar to the research results of Sellmer et al.^11^ The reason may be that the left ventricular volume and pressure load increased during the transition from fetal circulation to postpartum circulation, resulting in increased NT-proBNP secretion. Neonatal kidney clearance function was not mature, the clearance rate of NT-proBNP was reduced, leading to the early existence of physiological NT-proBNP increased phenomenon. With the decrease of vascular resistance, the decrease of pulmonary circulation pressure and the maturation of renal clearance, the level of NT-proBNP gradually decreased and gradually stabilized.^9^,^12^ The ROC curve analysis showed that the area under the ROC curve of PDA after three days of birth was 0.949, the critical value was 6,411pg/mL, the sensitivity was 92.3%, and the specificity was 92.9%. Such results suggested that serum NT-proBNP level was of a high predictive value on PDA of premature infants.^13^.^14^

## CONCLUSIONS

In this study, the serum NT-proBNP value of premature infants in the PDA group was higher than that in the aPDA group regardless of 3d or 5d after birth; ROC curve analysis showed that the serum NT-proBNP level of 3d and 5d after birth had both a predictive value for PDA High, the sensitivity and specificity of prediction are above 92%, suggesting that serum NT-proBNP levels at 3d and 5d after birth are helpful for early prediction of PDA. However, although this study shows that serum NTproBNP levels have a higher effect on PDA of premature infants High predictive value, but there is still a certain proportion of missed diagnosis rate and misdiagnosis rate, suggesting that the sample size needs to be increased for further research to determine the best diagnostic value of NT-proBNP for early prediction of PDA.

### Authors Contribution:

**CW:** Conceived the study, literature review, analysis of data and drafting of the paper.

**YS:** Helped in design, data collection, article drafting & critical revision.

**JJ:** Takes the responsibility and is accountable for all aspects of the work in ensuring that questions related to the accuracy or integrity of any part of the work are appropriately investigated and resolved.
